# Performance of universal adhesives on bonding to leucite-reinforced ceramic

**DOI:** 10.1186/s40824-015-0035-1

**Published:** 2015-05-22

**Authors:** Ryan Jin-Young Kim, Jung-Soo Woo, In-Bog Lee, Young-Ah Yi, Ji-Yun Hwang, Deog-Gyu Seo

**Affiliations:** Department of Conservative Dentistry and Dental Research Institute, School of Dentistry, Seoul National University, 101 Daehak-ro, Jongno-gu, Seoul, Korea; Dental Research Institute and School of Dentistry, Seoul National University, Seoul, Korea; Department of Dentistry, Inje University Seoul Paik Hospital, Seoul, Korea; Nutrition Education Major, Graduate School of Education, Sangmyung University, Seoul, Korea

**Keywords:** Leucite-reinforced ceramic, Microshear bond strength, Resin cement, Thermocycling, Universal adhesive

## Abstract

**Background:**

This study aimed to investigate the microshear bond strength of universal bonding adhesives to leucite-reinforced glass-ceramic.

**Methods:**

Leucite-reinforced glass-ceramic blocks were polished and etched with 9.5% hydrofluoric acid for 1 min. The specimens were assigned to one of four groups based on their surface conditioning (n = 16): 1) NC: negative control with no further treatment; 2) SBU: Single Bond Universal (3M ESPE); 3) ABU: ALL-BOND Universal (Bisco); and 4) PC: RelyX Ceramic Primer and Adper Scotchbond Multi-Purpose Adhesive (3M ESPE) as a positive control. RelyX Ultimate resin cement (3M ESPE) was placed on the pretreated ceramic and was light cured. Eight specimens from each group were stored in water for 24 h, and the remaining eight specimens were thermocycled 10,000 times prior to microshear bond strength evaluation. The fractured surfaces were examined by stereomicroscopy and scanning electron microscopy (SEM).

**Results:**

After water storage and thermocycling, the microshear bond strength values decreased in the order of PC > SBU and ABU > NC (P < 0.05). Thermocycling significantly reduced the microshear bond strength, regardless of the surface conditioning used (P < 0.05). Cohesive failure in the ceramic and mixed failure in the ceramic and resin cement were observed in the fractured specimens. The percentage of specimens with cohesive failure after 24 h of water storage was: NC (50%), SBU (75%), ABU (75%), and PC (87%). After thermocycling, the percentage of cohesive failure in NC decreased to 25%; however, yet the percentages of the other groups remained the same.

**Conclusions:**

Although the bond strength between resin and hydrofluoric acid-etched glass ceramic was improved when universal adhesives were used, conventional surface conditioning using a separate silane and adhesive is preferable to a simplified procedure that uses only a universal adhesive for cementation of leucite-reinforced glass-ceramic.

## Background

The considerable improvements made in adhesive dentistry and dental materials over the last few decades have seen non-metallic restorative materials such as ceramic being widely used in daily dental practice to keep up with increasing patient demands for esthetic treatment.

Dental ceramics can be broadly defined based on their composition as either silica-based or non-silica-based. This difference has pronounced clinical implications, as hydrofluoric acid treatment only modifies the microstructure of silica-based ceramics (glass ceramics) such as feldspathic porcelain, leucite-reinforced ceramic, and lithium-disilicate ceramic. Non-silica-based ceramics (polycrystalline ceramics) such as alumina and zirconia, on the other hand, are not affected [1].

To ensure an optimum bond with silica-based ceramics, the recommended surface conditioning prior to luting with a resin cement includes hydrofluoric acid etching to create a micromechanically retentive surface [2,3] This is followed by silane application to provide a chemical covalent and hydrogen bond [4,5] and finally the application of a bonding agent [6]. The bonding surface of the tooth substrate also needs to be conditioned by either self-etching with acidic monomer or total-etching with phosphoric acid, followed by priming and bonding. This conventional bonding procedure is not only time-consuming and technique sensitive, but also requires various products and armamentarium. Furthermore, because multiple procedures are required for different products, there is a greater tendency for error between steps if each procedure is not executed in accordance with the manufacturer’s recommendation.

A new type of bonding agent was recently introduced to help clinicians save chairtime and simplify the conditioning of both the tooth and restoration surface. This new generation of bonding agent has been named “universal”, as it can be used as a total-etch, self-etch or selective-etch adhesive. It is also capable of binding to the tooth structure, as well as indirect substrates such as ceramic, resin, and metal [7]. Of the bonding agents developed, Single Bond Universal adhesive (3M ESPE, St. Paul, MN, USA) and ALL-BOND Universal adhesive (Bisco, Schaumburg, IL, USA) are most commonly used. Evaluations of the effect of this universal bonding system on resin cement to indirect restorations have yielded promising results, with the universal adhesive bonding effectively to stainless steel [8], lithium disilicate [9], and zirconia [10]. From a clinician’s perspective, this new type of adhesive has been long awaited as a solution to the cumbersome multiple steps required for traditional adhesive systems.

Among the various methods developed for measuring the bond strength of an adhesive system, microshear bond strength (μSBS) testing [11] offers similar benefits as microtensile strength (μTBS) testing. That is, it provides a more evenly distributed stress through a reduced specimen size. Specimen preparation for the μSBS test is also less demanding than that for the μTBS test. There is, however, only very limited information available as to the effectiveness of a simplified bonding procedure using universal adhesives for the cementation of leucite-reinforced glass-ceramic to resin. This study therefore investigates the performance of universal bonding agents on leucite-reinforced glass-ceramic by measuring their μSBS before and after thermocycling.

## Methods

### Specimen preparation

**Figure 1:**
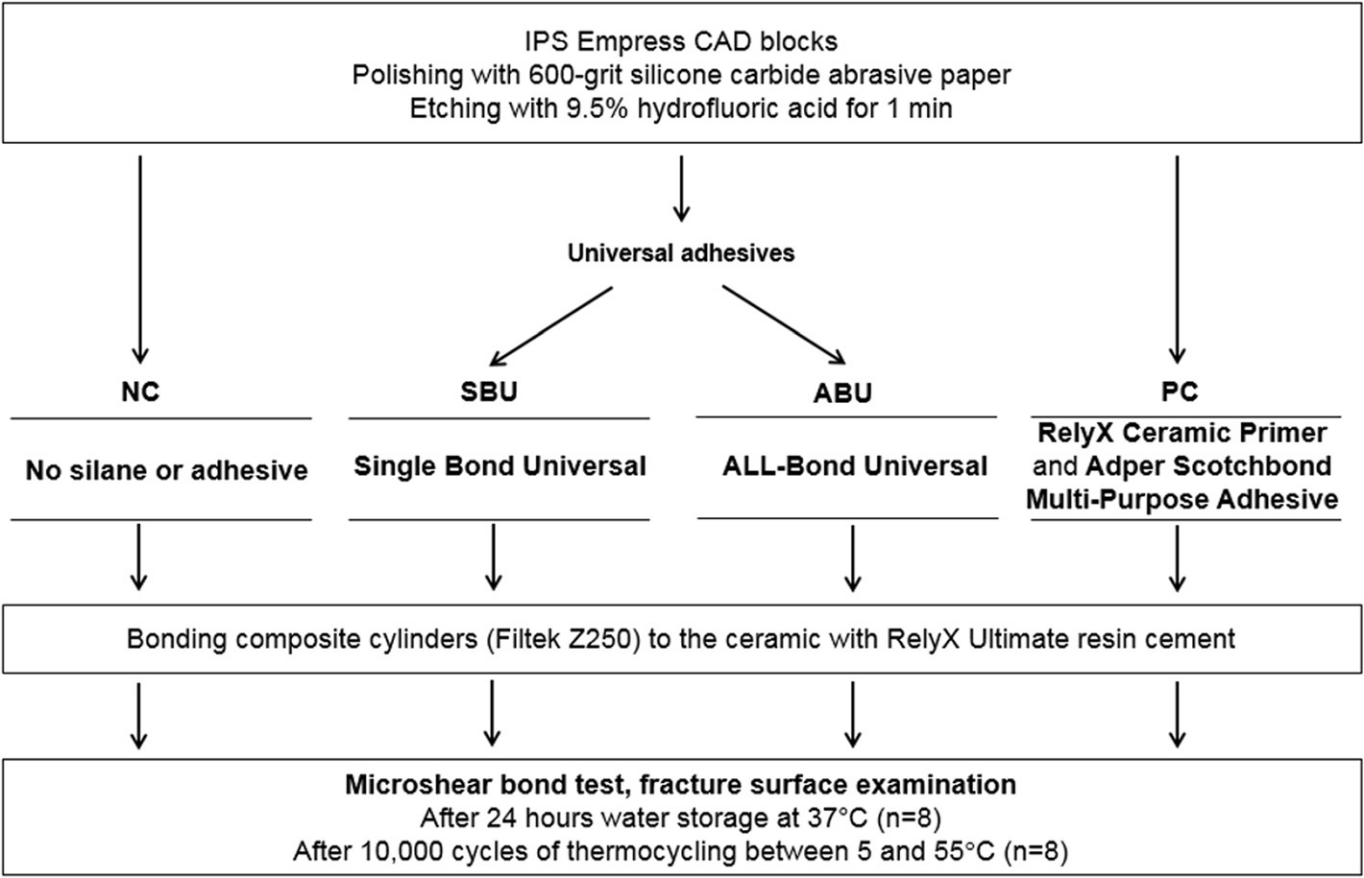
Experimental design of the study.

The name, composition and manufacturer of each material used are listed in Table 1, and a schematic of the experimental design is given in Figure 1. Leucite-reinforced glass-ceramic blocks (IPS Empress CAD, Ivoclar Vivadent, Schaan, Liechtenstein) were polished on up to 600-grit silicon carbide paper (Rotopol-V, Struers, Ballerup, Denmark) under running water. These blocks were then etched with 9.5% hydrofluoric acid (Porcelain etchant, Bisco) for 1 min, water rinsed, and cleaned by ultrasonication for 3 min in isopropyl alcohol. Each specimen was randomly assigned to one of four groups of sixteen according to the surface conditioning agents applied, as per the following:

**Table 1:** **Materials used in this study**

**Materials (Lot no.)**	**Composition**	**Manufacturer**
IPS Empress CAD (R04751)	Silicon dioxide, aluminum oxide, potassium oxide, sodium oxide, other oxides, pigments (Leucite-reinforced glass-ceramic)	Ivoclar Vivadent, Schaan, Liechtenstein
Porcelain etchant (9.5%) (120006991)	Hydrofluoric acid, polysulfonic acid	Bisco, Schaumburg, IL, USA
Single Bond Universal (539321)	MDP, Bis-GMA, HEMA, decamethylene DMA, ethanol, water, silane treated silica, 2-propenoic acid, −methyl-, reaction products with 1,10-decanediol and phosphorous oxide, copolymer of acrylic and itaconic acid, dimethylaminobenzoate(−4), camphorquinone, (dimethylamino)ethyl methacrylate, methyl ethyl ketone	3M ESPE, St. Paul, MN, USA
ALL-BOND Universal (1400002645)	MDP, Bis-GMA, HEMA, ethanol, water, initiators	Bisco, Schaumburg, IL, USA
RelyX Ceramic primer (N526043)	Ethyl alcohol, water, methacryloxypropyl-trimethoxysilane	3M ESPE, St. Paul, MN, USA
Adper Scotchbond Multi-Purpose Adhesive (N530683)	Bis-GMA, HEMA, triphenylantimony	3M ESPE, St. Paul, MN, USA
RelyX Ultimate (545247)	*Base*: Silane treated glass powder, 2-propenoic acid, 2-methyl-,1,1-[1-(hydroxymethyl)-1,2-ethanediyl] ester, reaction products with 2-hydroxy-1,3-propanediyl DMA and phosphorus oxide, TEGDMA, silane treated silica, oxide glass chemicals, sodium persulfate, tert-butyl peroxy-3,5,5-trimethylhexanoate, copper (II) acetate monohydrate *Catalyst*: Silane treated glass powder, substituted DMA, 1,12-dodecane DMA, silane treated silica, 1-benzyl-5-phenyl-barbic-acid, calcium salt, sodium p-toluenesulfinate, 2-propenoic acid, 2-methyl-, [(3-metoxypropyl) imino]di-2,1-ethanediyl ester, calcium hydroxide, titanium dioxide	3M ESPE, St. Paul, MN, USA

Abbreviations: Bis-GMA, bisphenol-A diglycidyl ether dimethacrylate; DMA, dimethacrylate; HEMA, hydroxyethyl methacrylate; MDP, 10-methacryloyloxydecyl dihydrogen phosphate; TEGDMA, triethylene glycol dimethacrylate.

- NC: Negative control with no further treatment.
- SBU: Single Bond Universal adhesive (3M ESPE) was applied for 20 s and air dried for 10 s.
  ABU: ALL-BOND Universal adhesive (Bisco) was applied for 20 s and air dried for 10 s.
- PC: Positive control. RelyX Ceramic Primer (3M ESPE) was applied for 20 s and air dried for 10 s. This was then followed by the application of a thin layer of Adper Scotchbond Multi-Purpose Adhesive (3M ESPE).

A dual-cured resin cement (RelyX Ultimate, 3M ESPE) was mixed and used to fill polyethylene tubes (Tygon R-3603 tubing, Saint-Gobain Co., Courbevoie, France), which were then placed over the ceramic specimen. These tubes were removed after light curing from four directions for 20 s per side at 800 mW/cm^2^ (Elipar FreeLight 2, 3M ESPE), leaving a resin cement cylinder with a diameter of 0.8 mm and a height of 1 mm.

### Microshear bond strength measurements

Eight specimens from each group were subjected to bond strength testing after storage in distilled water at 37°C for 24 h, while the remaining eight specimens in each group were thermocycled 10,000 times at 5 and 55°C (25 s dwell time) prior to testing. A universal testing machine (LF Plus, Lloyd Instruments, Fareham, UK) was used to apply a shear force through a 0.2 mm diameter stainless steel orthodontic wire positioned as close as possible to the bonded interface. This was moved at a crosshead speed of 0.5 mm/min until failure.

The data obtained were analyzed using one-way analysis of variance and the Tukey’s honest significant difference post-hoc test to evaluate any differences amongst the surface treatment conditioning protocols. The effect of thermocycling on the bond strength of each group was assessed using a paired-t test (SPSS software version 21, IBM, Armonk, NY, USA), in which an α level of 0.05 was considered statistically significant.

### Examination of the fractured surface

Following μSBS testing, the fractured surfaces of the ceramic blocks were examined with a stereomicroscope (SZ4045, Olympus Optical Co. Ltd., Tokyo, Japan) at 40x magnification to determine the precise failure mode. This was defined as either cohesive failure if the fracture occurred within the ceramic, or as mixed failure if the fracture occurred simultaneously within the ceramic and resin cement. The fractured specimens were also subjected to SEM examination at 200x magnification (S-4700 FESEM, Hitachi, Tokyo, Japan).

## Results

### Microshear bond strength

The mean and standard deviation of the μSBS (MPa) value for each group shown in Table 2 reveal that after storage in water for 24 h at 37°C, the μSBS decreased in the order of PC > SBU > ABU > NC. This order remained unchanged after 10,000 thermocycles; however, all groups did exhibit a significant reduction in μSBS after thermocycling (P < 0.05). Thus, regardless of thermocycling, the highest and lowest μSBS values were always observed in PC and NC, respectively (P < 0.05), while no significant difference was found between SBU and ABU (P > 0.05).

**Table 2:** **Mean and standard deviation (SD) of micro-shear bond strength (in MPa)**

**Group**	**Water storage (24 hour)**	**Thermocycling (10,000 cycles)**
NC	22.71 (2.22)^c^	16.60 (4.37)^c,^*
SBU	27.99 (3.89)^b^	23.89 (2.00)^b,^*
ABU	27.22 (2.06)^b^	22.76 (3.90)^b,^*
PC	32.92 (3.41)^a^	27.91 (3.05)^a,^*

Within the same column, values with different superscript lower case letters are statistically significantly different (Tukey HSD, P < 0.05).

*indicates significant reduction in bond strength of each group after 10,000 thermocycles (Paired T-test, where P < 0.05).

Abbreviations: NC, negative control; SBU, Single Bond Universal; ABU, ALL-BOND Universal; PC, positive control.

### Failure mode

**Figure 2:**
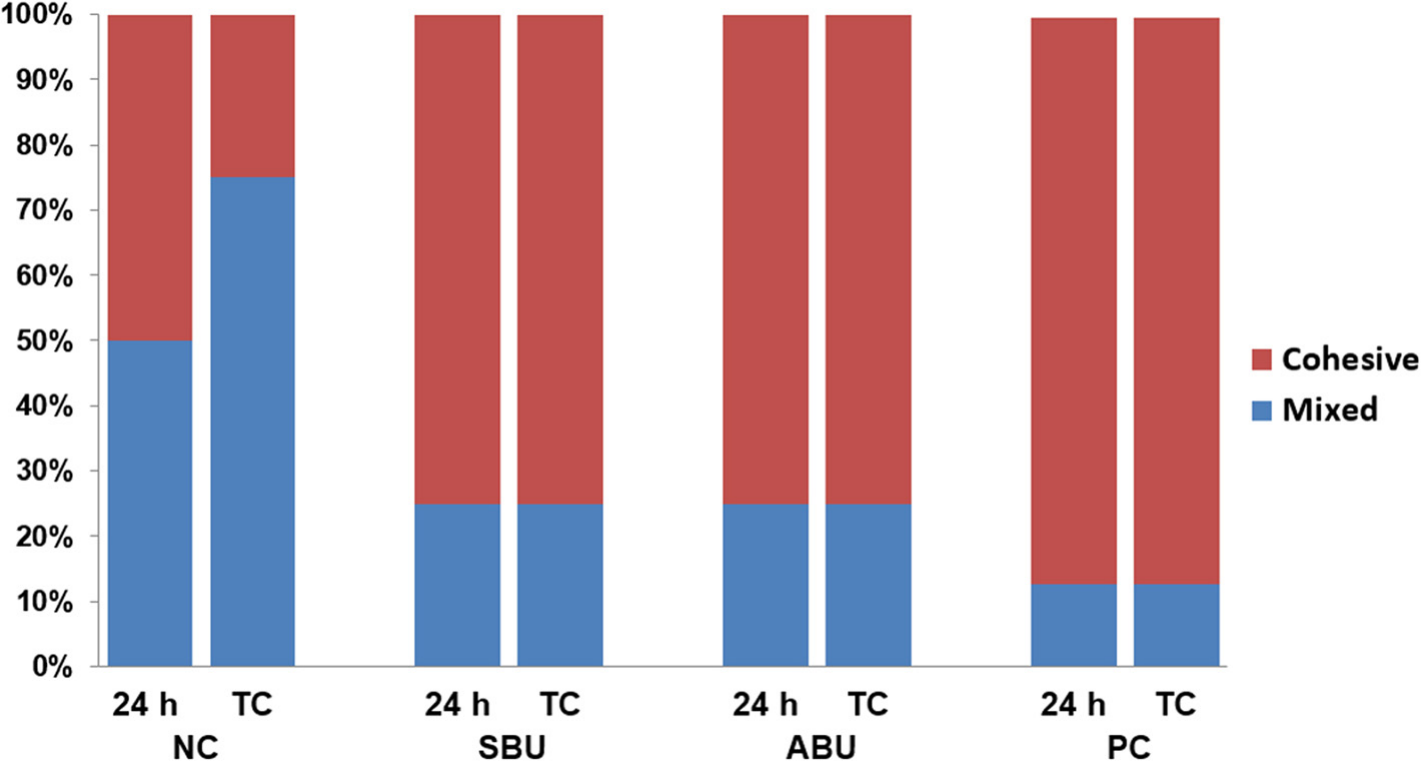
Percentage distribution of failure modes after 24 h and 10,000 thermocycles. Abbreviations: NC, negative control; SBU, Single Bond Universal; ABU, ALL-BOND Universal; PC, positive control.

**Figure 3:**
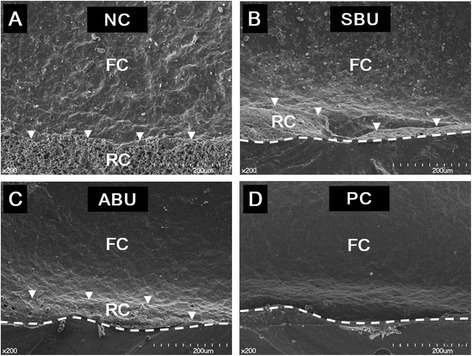
Representative SEM images of the fractured ceramic specimens. **(A)** Within the cemented area in NC (negative control), **(B-D)** At the interface between the initial cemented surface (area above the dashed lines) and uncemented surface of the ceramic in SBU (Single Bond Universal), ABU (ALL-BOND Universal), and PC (positive control), respectively. Arrowheads **(A-C)** indicate the junction between the resin cement (RC) and fractured ceramic (FC). Magnification x200.

The distribution of failure mode for each group in Figure 2 reveals a predominance of cohesive failure within the ceramic in all of the surface conditioned groups. After 24 h of water storage, the number of specimens with cohesive and mixed failure modes in each group was: NC (4:4), SBU (6:2), ABU (6:2), and PC (7:1). Thermocycling produced no discernible change in the failure mode distribution except in the case of NC, which exhibited 2 cohesive and 6 mixed failure modes. In the representative SEM images from each group presented in Figure 3, varying degrees of cohesive ceramic failure can be observed in all fractured surfaces. Deeper and larger areas of cohesive ceramic fracture, in which there are fewer resin cement remnants, are also more frequently observed in the conditioned ceramic specimens in SBU, ABU, and PC than in the unconditioned ceramic of NC.

## Discussion

The performance of the two universal adhesives with regards to the μSBS at the interface between leucite-reinforced glass-ceramic and resin cement was found to be highest in the positive control, wherein a traditional bonding technique was used that entailed the separate application of silane and adhesive. The use of silane has been widely recommended as a way of maximizing the bonding of silica-based ceramic to resin [4,5,12-14], as their hydroxyl and organofunctional terminal groups are capable of bonding with silica and resin, respectively [14,15].

The main difference in the composition of the two universal adhesives is the incorporation of silane, which is only present in Single Bond Universal. Therefore SBU was expected to produce a greater bond strength than ABU; however, the μSBS values obtained for each were the same. This implies that the silane contained in SBU failed to produce any significant chemical bonds with the ceramic, especially considering the lower bond strength compared to the positive control. This finding corroborates the results of Kalavacharla *et al*. [9], who compared the effect of Single Bond Universal with and without saline application on lithium-disilicate bond strength. They reported that the bond strength was significantly improved when silane was applied prior to the application of the universal adhesive; thus, the incorporation of silane in the universal adhesive itself would seem ineffective in improving the ceramic-resin bond. This could be explained by the presence of a mixture of various components within the same bottle, as it has been reported that bis-GMA may inhibit the action of silane by disrupting the condensation reaction with the hydroxyl group of a silica-based ceramic [16]. Furthermore, the acidic functional monomer 10-MDP (methacryloyloxydecyl dihydrogen phosphate) that is incorporated in universal adhesives may impede the ideal chemical interaction between silane and ceramics owing to the tendency for premature hydrolysis in an acidic environment [14]. Nevertheless, MDP is a proven bifunctional adhesive monomer that can bind to metal [17] or zirconia [18-20], with a hydrophilic phosphate terminal end that chemically binds to oxides and a hydrophobic methacrylate terminal end that copolymerizes resin monomers [21].

The results also show that despite having the lowest bond strength, the negative control offers a substantial resistance against shear force. This can be attributed to the increased retentive surface area created through preferential dissolution of the glassy phase in the silica-based ceramic during hydrofluoric acid etching [22]. Notably, when compared to the conditioned groups, the negative control tended to produce a shallower and smaller area of cohesive ceramic fracture with a greater number of resin cement remnants. Furthermore, the negative control experienced a greater level of reduction in bond strength after thermocycling (28%) when compared to the conditioned groups (15–16%). The application of universal adhesives (SBU and ABU) yielded a significantly greater bond strength than the negative control; this is likely because their lower viscosity ensures a greater filling of the micro-irregular etched surface and greater micromechanical retention.

Within the limitations of this study, the highest bond strength was achieved by the application of a separate silane and adhesive. This means that despite the benefits of universal bonding in terms of simplicity, convenience, cost-effectiveness, and clinically acceptable bond strength, traditional surface conditioning procedures still provide a more durable bond between silica-based ceramics and resin cement. The caveat here, however, is that achieving such a result with traditional techniques requires that the clinical steps for each product be strictly followed.

## Conclusions

Universal adhesives significantly improve the bond strength between a resin and hydrofluoric acid-etched glass ceramic. However, for the most durable bond, a conventional surface conditioning procedure using a separate silane and adhesive is preferable for the cementation of leucite-reinforced glass-ceramic than a simplified procedure using a universal adhesive alone. Regardless of the surface conditioning procedure used, thermocycling significantly reduces the μSBS, especially when the ceramic surface is not conditioned.

### Availability of supporting data

The data sets supporting the results of this article are included within the article.
